# Infectious spleen and kidney necrosis virus induces the reactive oxidative species/Nrf2-mediated oxidative stress response for the regulation of mitochondrion-mediated Bax/Bak cell death signals in GF-1 cells

**DOI:** 10.3389/fmicb.2022.958476

**Published:** 2022-10-11

**Authors:** Pin-Han Chen, Tsai-Ching Hsueh, Jiann-Ruey Hong

**Affiliations:** ^1^Lab of Molecular Virology and Biotechnology, Department of Biotechnology and Bioindustry Sciences, Institute of Biotechnology, National Cheng Kung University, Tainan City, Taiwan; ^2^Institute of Biotechnology, National Cheng Kung University, Tainan City, Taiwan

**Keywords:** ISKNV, Nrf2-mediated oxidative stress, BAX/BAK, antioxidant, DNA virus, viral treatment

## Abstract

Infectious spleen and kidney necrosis virus (ISKNV) infections can trigger host cell death and are correlated with viral replication; however, they have rarely been considered in terms of the host organelle involvement. In the present study, we demonstrated that ISKNV triggered an oxidative stress signal in the Nrf2-mediated oxidative stress response and induced stress signals for Bax/Bak-mediated host cell death in fish GF-1 cells. The results showed that after ISKNV infection, the levels of reactive oxidative species (ROS) increased by 60–80% from day 3 to day 5, as assessed by an H2DCFDA assay for tracing hydrogen peroxide (H_2_O_2_), which was correlated with up to a one-fold change in the fish GF-1 cells. Furthermore, we found that ISKNV infection induced Nrf2-mediated ROS stress signals from D1 to D5, which were correlated with the upregulation of antioxidant enzymes, such as catalase, SOD1, and SOD2; these effects were blocked by the antioxidants GSH and NAC. By analyzing Nrf2-mediated ROS stress signals for cell death regulation *via* an apoptotic assay, we found that treatment with antioxidants reduced annexin-V-positive signals by 10% (GSH) to 15% (NAC); moreover, necrotic-positive signals were reduced by 6% (GSH) and 32% (NAC) at day 5 (D5) in GF-1 cells, as indicated by PI staining. Furthermore, we found that Nrf2-mediated ROS stress regulated mitochondrion-mediated Bax/Bak death signals at D3 and D5; this was effectively blocked by antioxidant treatment in the GF-1 cells, as demonstrated by a JC1 assay (ΔΨm) and western blot analysis. In addition, we found that downstream signals for caspase-9 and -3 activation were apparently blocked by antioxidant treatment at D3 and D5. Finally, we found that treatment with GSH and NAC reduced major capsid protein (MCP) expression and virus titer (TCID_50%_) by up to 15-fold at D5 in GF-1 cells. Thus, our data suggest that ISKNV can induce ROS production, which triggers Nrf2-mediated stress signals. Then, these stress signals can regulate mitochondrion-mediated Bax/Bak apoptotic signaling, which is connected to downstream caspase-9 and -3 activation. If ISKNV-induced Nrf2-mediated stress signaling is blocked, then the antioxidants GSH and NAC can also suppress apoptotic signals or reduce viral replication. These findings may provide insights into the control and treatment of double-stranded DNA viruses.

## Introduction

Iridoviruses, which have caused a serious decline in the global population of amphibians (up to 20 species) and heavy economic losses in the aquaculture industry (Kurita and Nakajima, 2012; Subramaniam et al., 2012), belong to a large dsDNA virus family that includes five genera, namely, *Chloriridovirus, Ranavirus, Iridovirus, Lymphocystivirus*, and *Megalocytivirus* (Williams et al., 2005). These viruses are particles with icosahedral symmetry and a diameter of 120–200 nm. Megalocytiviruses infect a wide range of freshwater fish and tropical marine fish, including groupers, red sea bream, sea bass, gourami, angel fish, cichlids, and lampeyes, and cause very similar diseases in each species. The spleen, brain, kidneys, and gastrointestinal tract have been found to be the most targeted tissues during ISKNV infections in fish. In addition, it is sometimes found in the gills, liver, heart, and connective tissues (Huang et al., 2011; Pham et al., 2012), and its presence is linked to a high rate of mortality in the fish industry (Huang et al., 2011; Kurita and Nakajima, 2012; Subramaniam et al., 2012). Thus, in the study of tissue damage induced by ISKNV infection, the development of new and effective prevention strategies to block viral infection should be a focus of future research.

Oxidative stress has been implicated in the pathogenesis of neurodegenerative diseases, such as Alzheimer’s disease and Parkinson’s disease (Taylor et al., 2002; Jellinger, 2003), and coronavirus infections (Duca et al., 2021). Oxidative stress occurs in cells when the production of reactive oxygen species (ROS) exceeds the cell’s endogenous antioxidant defenses (Krapfenbauer et al., 2003). The two major cellular defenses against ROS are superoxide dismutases (SODs) and catalase (Desagher et al., 1996). SODs catalyze the dismutation of superoxide (O_2_^–^) to hydrogen peroxide (H_2_O_2_) and molecular oxygen (O_2_) and are located in the cytoplasm (Cu/ZnSOD; SOD1) and mitochondria (MnSOD; SOD2) (Shull et al., 1991). Catalase is a tetrameric iron porphyrin protein found in peroxisomes that scavenges H_2_O_2_, producing H_2_O and O_2_ (McClung, 1997). The expression of catalase and Cu/Zn SOD is constitutive, while MnSOD expression is induced by oxidant stress (Wang et al., 2003). Few studies have investigated the correlation between RNA virus-induced oxidative stress and diseases. While ROS are now known to be important secondary messengers, it is unclear whether they can regulate viral replication, even in HIV (Muller, 1992), HCV (de Mochel et al., 2010), and coronavirus (Duca et al., 2021) systems.

Intrinsic apoptosis is a form of programed cell death (RCD) initiated by a variety of microenvironmental perturbations, including (but not limited to) growth factor withdrawal, DNA damage, endoplasmic reticulum (ER) stress, reactive oxygen species (ROS) overload, replication stress, microtubular alterations, and mitotic defects (Nunez et al., 1990; Brumatti et al., 2010; Czabotar et al., 2014; Roos et al., 2016; Pihan et al., 2017; Vitale et al., 2017).

Intrinsic apoptosis (type I cell death) (Galluzzi et al., 2018) is mediated by the Bcl-2 family pro-apoptotic and anti-apoptotic proteins, which promote and inhibit death signals, respectively. Common members of the anti-apoptotic Bcl-2 family include Bcl-2, Bcl-xl, Mcl-1, and A1, which regulate key checkpoints in the apoptotic pathway (Ameisen, 2002; Kumar, 2006; Clarke and Tyler, 2009). On the other hand, the pro-apoptotic Bcl-2 family includes Bax, Bad, and Bik, which trigger death signals within cells that are connected to downstream signals for the activation of the caspase family. These apoptotic death signals facilitate the late phase of apoptosis, characterized by the cleavage of cellular proteins and the destruction of cellular structures.

Bcl-2 family proteins are subdivided into three groups on the basis of their pro- or anti-apoptotic action and Bcl-2 homology (BH) domains (Ameisen, 2002; Kumar, 2006; Youle and Strasser, 2008; Clarke and Tyler, 2009). Anti-apoptotic Bcl-2-like proteins (e.g., Bcl-2, Bcl-xL, Bcl-w, Mcl-1, and A1/Bfl-1) and pro-apoptotic Bax-like proteins (e.g., Bax, Bak, and Bok/Mtd) display four BH domains. On the other hand, pro-apoptotic BH3-only proteins (e.g., Bid, Bim/Bod, Bad, Bmf, Bik/Nbk, Blk, Noxa, Puma/Bbc3, and Hrk/DP5) possess only a short motif called the BH3 domain, as their name indicates. BH3 proteins integrate and transmit death signals that emanate from defective cellular processes to other Bcl-2 family members. Through their BH3 domain, these proteins can interact with anti-apoptotic proteins to inhibit their function and/or multidomain proteins, such as Bax and Bak, to stimulate their activity. The former are often referred to as “sensitizers,” while the latter are classified as “activators”. The multidomain pro-apoptotic proteins Bax and Bak, and perhaps Bok in some tissues, are responsible for mitochondrial outer membrane permeabilization (MOMP) and are the master effectors of apoptosis, as evidenced by the failure of cells lacking Bax and Bak to undergo MOMP and apoptosis in response to many death stimuli.

The mechanisms for the activation of pro-apoptotic Bcl-2 family members, such as Bax and Bad, have been well-studied. The activation of the pro-apoptotic Bcl-2 family protein Bax results from a highly regulated multistep process involving its translocation from the cytosol to the outer mitochondrial membrane (OMM), where it inserts itself and oligomerizes. In contrast to Bax, Bak is constitutively inserted into the OMM by a C-terminal transmembrane domain. Its insertion can be facilitated by the voltage-dependent anion channel isoform 2, with which it has been found to interact.

Typical apoptotic and non-apoptotic cell death has been examined with regard to iridovirus infection (Pham et al., 2012). In one study, GSIV serine/threonine kinase overexpression was found to induce apoptotic cell death, and its function was inhibited during Bcl-2/Bcl-xL overexpression in fish cells (Chen et al., 2016). From the molecular regulation perspective of megalocytivirus infections, especially the ISKNV strain, studies on the molecular mechanisms of fish host cell death are very limited.

In recent years, ISKNV has been found to induce Bax/Bak-mediated cell death by strongly interacting with the anti-apoptotic members Bcl-2 and Bcl-xL in the mitochondria from the early to late replication stages in GF-1 cells. The Bax/Bak-mediated cell death signal has been shown to be connected to downstream death signaling by caspase-9/caspase-3 activations in fish host cells (Chen et al., 2022). Examining cell death in terms of the molecular regulation and physiologic functions of DNA viruses, such as human cytomegalovirus (HCMV) and RNA viruses, such as influenza, which also contribute to host and pathogen interactions, will provide a better understanding of molecular pathogenesis in diseases and lead to better therapeutic strategies for disease control (Hong et al., 2005; Labbe and Saleh, 2008; Chen et al., 2020; Shiu et al., 2018). Thus, a more in-depth understanding of the molecular mechanisms behind the ISKNV infection-induced cell death pathway is urgently required.

Studies on the induction of mitochondrion-mediated cell death *via* ISKNV infection, which is linked to viral replication, are limited. In this study, we found that ISKNV induced ROS/Nrf2-mediated stress signals upon intrinsic apoptotic cell death *via* a Bax/Bak-mediated death pathway. In addition, we found that drug treatment using antioxidants could effectively suppress the ROS/Nrf2-mediated stress signal and reduce intrinsic apoptotic cell death. These findings provide novel insights into iridovirus-regulated molecular pathogenesis and treatment strategies.

## Materials and methods

### Chemicals, drugs, and antibodies used

The experiments required the use of the MitoCapture reagent (Mitochondria BioAssayTM Kit; BioVision, Mountain View, CA, United States) and an annexin-V-fluorescein assay (Boehringer-Mannheim, Mannheim, Germany). The following antibodies were used: anti-mouse Bax MAb (Cell signaling Tech., Code No. #2772), anti-mouse Bak MAb (Cell signaling Tech., Code No. #12105), anti-mouse Nrf2 MAb (ENZO, Code No. Q16236), anti-mouse catalase MAb (Rockland, Code No. 200-401-051), anti-mouse Cu/ZnSOD MAb (Cayman Chemical, Code No. 10011388), anti-mouse MnSOD MAb (GeneTex, GTX 116093), anti-mouse β-actin (Millipore), anti-mouse caspase-3 MAb (Cell signaling Tech., Code No. #9662), and anti-mouse caspase-9 MAb (Cell Signaling Tech., Code No. #9508). The following compounds were also used: GSH (Glutathione; Sigma, Catalog No. G4251), NAC (*N*-acetylcysteine; Sigma, Catalog No. A7250), Sigma, Catalog No. G4251), and NAC (*N*-acetylcysteine; Sigma, Catalog No. A7250).

### Cell and virus cultures

Grouper fin cells (GF-1 cells) were provided by Dr. Chi (Institute of Zoology and Development of Life Sciences). The cells were maintained under standard conditions (at 28°C) in Leibovitz’s L-15 medium supplemented with 5% fetal bovine serum (FBS) and 25 μg/ml gentamycin antibiotic. The cells were passaged twice weekly. All the experiments were performed with cells in the logarithmic growth phase. Naturally infected Pagrus major (red seabream) were collected in 2016 from the Kaohsiung Prefecture; these fish were the source of RSIV-KU (accession number: Kt781098) that was used to infect the GF-1 cells used in this study. RSIV-KU is an ISKNV-like strain that has had its complete viral genome sequenced (Shiu et al., 2018; Chen et al., 2022). The virus was purified and stored at −80°C until use. The viral titer was determined using a median tissue culture infective dose (TCID_50_) assay, in accordance with the method developed by Dobos et al. (1979).

### Annexin V-FLUOS staining

An analysis of phosphatidylserine (PS) on the outer leaflet of the apoptotic cell membranes was performed using annexin V-fluorescein and propidium iodide (PI) to differentiate apoptotic from necrotic cells. At the end of various incubation times (0, 1, 2, 3, 4, and 5 days), each sample was removed from the medium and washed with PBS; then, the cells were incubated with 100 μl of staining solution (annexin V-fluorescein in a HEPES buffer containing PI; Boehringer-Mannheim, Mannheim, Germany) for 10–15 min. The evaluation was performed using fluorescence microscopy with an excitation wavelength of 488 nm and a 515 nm long-pass filter for detection (Hong et al., 1999; Chen et al., 2022).

### Evaluation of mitochondrial membrane potential using a lipophilic cationic dye

Changes in the mitochondrial membrane potential that occurred during ISKNC-induced apoptosis were examined using the JC1 Mito-ID membrane potential aggregation dye. The GF-1 cells were seeded at 1 × 10^5^ cells per ml on a 60 mm Petri dish for at least 20 h prior to cultivation. Then, the resulting monolayers were rinsed twice with PBS, after which the cells were infected with the virus or the antioxidant treatment was administered (2 mM GSH and 2 mM NAC) at an MOI of one before incubation for 0, 1, 2, 3, 4, and 5 days. At different time points after infection, the cells were washed, fixed, and permeabilized with PBS containing 0.2% Triton X-100-PBS for 5 min on ice. Then, they were incubated with Mito-ID JC1 dye for 30 min at 28°C, washed with PBS, suspended in LB medium, and analyzed using fluorescence microscopy (Chen et al., 2007, 2022). The evaluation was performed using fluorescence microscopy with an excitation wavelength of 488 nm and a 515 nm long-pass filter for detection.

### Western blotting analysis

The GF-1 cells were seeded at 1 × 10^5^ cells per ml on a 60 mm Petri dish for at least 20 h prior to cultivation. Then, the resulting monolayers were rinsed twice with PBS, after which the cells were infected with the virus or the antioxidant treatment was administered (2 mM GSH and 2 mM NAC) at an MOI of 1 before incubation for 0, 1, 2, 3, 4 and 5 days. The resulting monolayers were rinsed twice with PBS, after which the cells were infected with the virus at an MOI of 1 and incubated for 0–5 days. The culture media were aspirated at the end of each time point, after which the cells were washed with PBS and lysed in 0.3 ml of lysis buffer (10 mM Tris base, 20% glycerol, 10 mM sodium dodecyl sulfate, and 2% ß-mercaptoethanol; pH 6.8). The proteins were separated *via* sodium dodecyl sulfate polyacrylamide gel electrophoresis, electroblotted, and then subjected to immunodetection, as previously described (Hong et al., 1999; Chen et al., 2022). The membranes were incubated with a dilution of anti-mouse Bax MAb (1:1,000; Cell signaling Tech., Code No. #2772), anti-mouse Bak MAb (Cell signaling Tech., Code No. #12105), anti-mouse caspase-3 MAb (1:1,000, Cell signaling Tech., Code No. #9662), anti-mouse caspase-9 MAb (1:2,500; Cell Signaling Tech., Code No. #9508), anti-mouse Nrf2 MAb (1:2,500; ENZO, Code No. Q16236), anti-mouse catalase MAb (1:2,500; Rockland, Code No. 200-401-051), anti-mouse Cu/ZnSOD MAb (1:2,500; Cayman Chemical, Code No. 10011388), anti-mouse MnSOD MAb (GeneTex, GTX 116093), or anti-mouse ß-actin MAb (1:2,500; Chemicon, Temecula, CA, USA) along with a dilution of the appropriate secondary antibodies (1:7,500 to 1:10,000), which included peroxidase-labeled goat anti-mouse (Amersham, Piscataway, NJ, USA) and goat anti-rabbit (Amersham) antibodies. The detection of chemiluminescence was performed using a Western Exposure Chemiluminescence Kit (Amersham) according to the manufacturer’s instructions. The presence of antibody binding was determined using a Top Bio Multigel-21.

### Statistical analyses

The loss of MMP and the percentage of annexin V-fluorescein-positive cells/PI staining were determined in each sample by counting 200 cells. Each result is expressed as the mean S.E. The data were analyzed using either paired or unpaired Student’s *t*-tests, as appropriate. The quantification of these results was performed for three individual experiments. A value of *p* < 0.05 was taken to indicate a statistically significant difference between the group mean values (Chen et al., 2022).

## Results

### Infectious spleen and kidney necrosis virus induces reactive oxidative species production and enhanced viral titers in GF-1 cells

**FIGURE 1:**
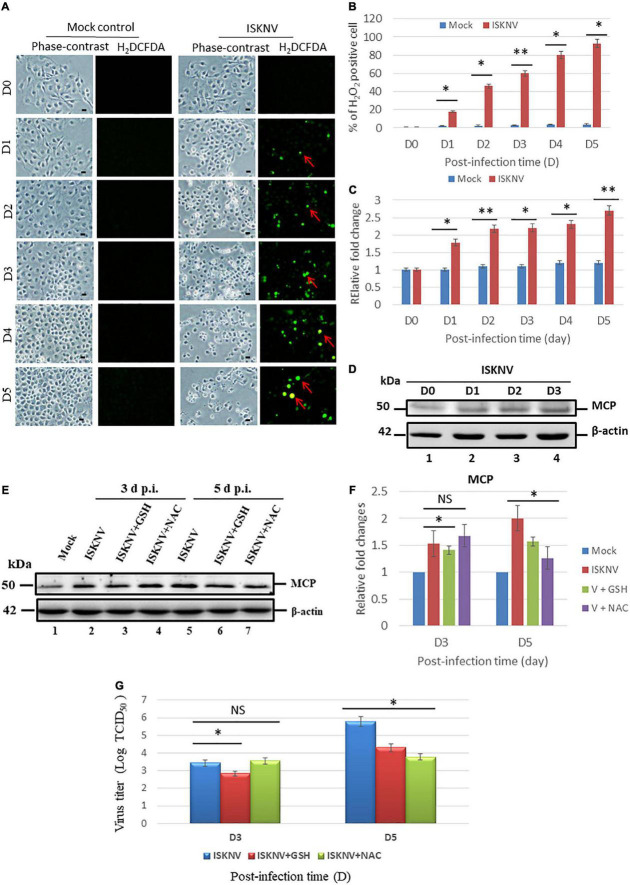
Infectious spleen and kidney necrosis virus (ISKNV) can induce reactive oxidative species (ROS) production and is correlated with viral replication in fish GF-1 cells. **(A)** Phase-contrast/green fluorescence micrographs of ISKNV-infected fish cells from day 0 to day 5. Phase contrast images of rounded-up cells were stained with H_2_DCFDA to examine ROS production: positive cells are indicated by red arrows. Scale bar = 10 μm. **(B)** Percentage of H_2_DCFD (green fluorescence)-positive ISKNV-infected cells from day 0 to day 5. The quantification of these results was performed for three individual experiments. All data were analyzed using either paired or unpaired Student’s *t*-tests, as appropriate. *P < 0.01. **(C)** Relative fold changes in ROS production according to the H_2_DCFD assay of ISKNV-infected cells at different time points. Day 0 values from the mock group were used as the normal control and set at one-fold. The number of virus-infected cells in each of the three images was counted in three individual experiments using the Image J software. All data were analyzed using either paired or unpaired Student’s *t*-tests, as appropriate. *P < 0.01. **(D)** Western blot analysis of ISKNV major capsid protein expression in fish cells following infection and incubation on day 0 (lane 1), day 1 (lane 2), day 2 (lane 3), and day 3 (lane 4). MCP proteins were detected using western blot analysis; the gels were immunoblotted with a polyclonal antibody for the ISKNV major capsid protein and ß-actin was used as an internal control. **(E)** Identification of viral MCP suppression by antioxidants *via* western blot analysis. GF-1 cells infected with ISKNV and treated with GSH (2 mM) and NAC (2 mM) are shown at day 0 (mock control), day 3, and day 5, where lanes 1–7 correspond to ISKNV-infected fish cells. The blots were probed with an anti-rabbit MCP polyclonal antibody (1:5,000) and an anti-mouse β-actin monoclonal antibody (1:12,500). **(F)** Quantification of MCP protein expression level (*N* = 3) using the Image J software, as shown in panel **(E)**. All data were analyzed using either paired or unpaired Student’s *t*-tests, as appropriate. *P < 0.01. **(G)** Quantification of ISKNV viral titer *via* TCID_50_ assay (*N* = 3), as shown in panel **(A)**. All data were analyzed using either paired or unpaired Student’s t-tests, as appropriate. *P < 0.01, **P < 0.05. ns, no significant different.

The role of ISKNV infection in ROS generation showed that it was correlated with cell destruction. In the ROS generation analysis using an H_2_DCFDA assay, we found that ISKNV increased ROS production from D1 to D5 (Figure 1A) when compared to mock-control group, indicating hydrogen peroxide (H_2_O_2_) generation. We counted the ROS-positive cells using fluorescence microscopy and found an increase from D1 (18%) to D5 (94%) when compared to D0 (0.2%), as shown in Figure 1B. ROS generation was also analyzed using the Image J program to determine relative fold changes from D1 (1.8-fold) to D5 (2.7-fold) compared with the D0 control (set as one-fold), as shown in Figure 1C. Viral protein expression was determined based on MCP expression (Figure 1D). The monitored time points were D0, D1, D2, and D3. The expression level of MCP gradually increased from D1 to D3 and was correlated with ROS generation in host cells.

To determine whether ISKN-mediated ROS production stress signals could modulate viral expression, the MCP protein was investigated using western blot analysis. According to the results, ISKNV-induced ROS/Nrf2-mediated stress signals suppressed MCP expression at D5 in the GSH (Figure 1E, lane 6) and NAC (Figure 1E, lane 7) treatment groups compared with the ISKNV-infected group (Figure 1E, lane 5). Expression was also quantified *via* an estimation using densitometry in terms of relative fold changes, as shown in Figure 1F. At D3, an up to 0.5-fold change was observed in the NAC group; at D5, both the GSH and NAC groups showed up to a 0.4-fold change. On the other hand, MCP suppression was very dynamic at D3 and was reduced by up to 0.3-fold; however, at D5, GSH showed a 0.4-fold change and NAC showed a 0.8-fold change, which were also correlated with the viral titer assay using TCID_50_ (Figure 1G).

### Antioxidants can block reactive oxidative species production in infectious spleen and kidney necrosis virus-infected GF-1 cells

**FIGURE 2:**
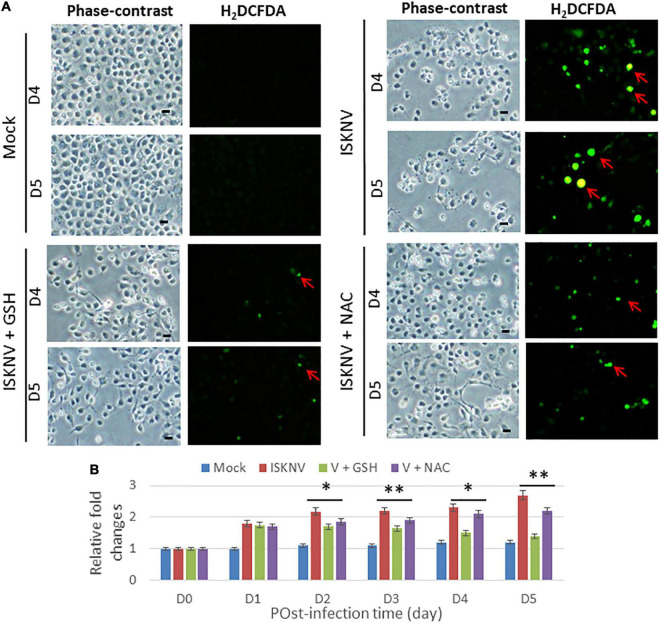
Blockage of infectious spleen and kidney necrosis virus (ISKNV) induces reactive oxidative species (ROS) production *via* antioxidants in fish cells. **(A)** Phase-contrast/green fluorescence micrographs of ISKNV-infected and GSH- (2 mM) and NAC (2 mM)-treated fish cells at day 4 and day 5. Phase contrast images in which rounded-up cells were stained with H_2_DCFDA to examine ROS production: positive cells are indicated by red arrows. Scale bar = 10 μm. **(B)** Relative fold changes in ROS production determined *via* an H_2_DCFD assay of ISKNV-infected cells at different time points. Day 0 of the mock group was used as the normal control and set at one-fold. The counting of virus-infected cells in each of the three images was repeated in three individual experiments using the ImageJ software. All data were analyzed using either paired or unpaired Student’s *t*-tests, as appropriate. *P < 0.01 and **P < 0.05.

Next, we tested the antioxidants GSH and NAC for their ability to block ROS production during ISKNV infection in GF-1 cells using an H_2_DCFDA assay. With regard to the reduced efficacy of ISKNV-infected groups, we found that they could apparently suppress ROS production from D4 to D5 (Figure 2A) when compared to the mock-treated group. Figure 2B shows that ROS generation was reduced in terms of relative fold changes from D4 (0.8-fold) to D5 (1.3-fold) in the GSH-treated group and at D4 (0.3-fold) to D5 (0.5-fold) in the NAC-treated group compared with the ISKNV-infected group at D4 (2.3-fold) to D5 (2.7-fold) and the mock group at D4 (1.3-fold) to D5 (1.3-fold). Regarding the blocking efficacy of the antioxidants, we found that GSH was superior to NAC.

### Infectious spleen and kidney necrosis virus induces the Nrf2-mediated stress response and the expression of leading antioxidant enzymes in GF-1 cells

**FIGURE 3:**
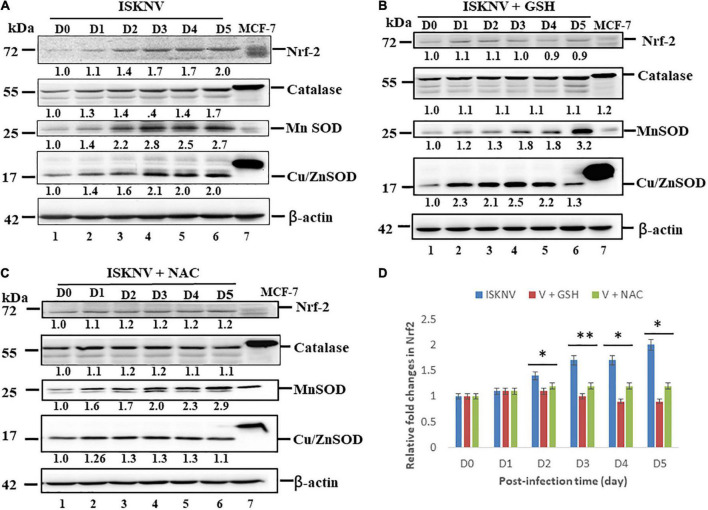
Infectious spleen and kidney necrosis virus (ISKNV) infection can induce Nrf2 and antioxidant enzyme expression in GF-1 cells. **(A–C)** Infection of fish cells with ISKNV from day 0 to day 5, showing ISKNV-infected cells **(A)**, GSH-treated and virus-infected cells **(B)**, and NAC-treated and virus-infected cells **(C)**, with the stress transcriptional factor Nrf2 and the antioxidant enzyme catalases Cu/MnSOD and ZnSOD, as determined by western blot analyses. Lanes 1–6 correspond to ISKNV-infected fish cells from day 0 to day 5, while lane 7 shows the MCF7 cell lysate. Membranes were probed with anti-mouse Nrf2, catalase, Cu/MnSOD, and ZnSOD monoclonal antibodies (1:7,500) and an anti-mouse β-actin monoclonal antibody (1:12,500). **(D)** Quantification of protein and Nrf2 expression levels (*N* = 3) using the Image J software, from panels **(A–C)**. All data were analyzed using either paired or unpaired Student’s *t*-tests, as appropriate. *P < 0.01 and **P < 0.05.

To determine whether ISKNV could induce ROS-mediated stress signals, stress signals were screened using the transcriptional factor Nrf2 and the antioxidant enzyme catalases MnSOD (SOD2) and Cu/ZnSOD (SOD1) *via* western blot analysis. According to the results, ISKNV-induced ROS-mediated stress signals quickly upregulated Nrf2 and the antioxidant enzyme catalases MnSOD and Cu/ZnSOD from D1 (Figure 3A, lane 2 and Supplementary Figure 1A) to D5 (Figure 3A, lane 6 and Supplementary Figure 1A) when compared to D0 as a normal control (as shown in Figure 3A, lane 1 and Supplementary Figure 1A). We also found that antioxidant treatment with GSH (2 mM) and NAC (2 mM) decreased hydrogen peroxide production and reduced the induction of stress signals. In addition, we observed that GSH and NAC blocked Nrf2, catalase, and Cu/ZnSOD upregulation, showing a dynamic trend from D1 to D5, but did not inhibit MnSOD at D5. Next, we analyzed Nrf2 expression levels (Figure 3D) in the GSH-treated group (Figure 3B and Supplementary Figure 1B) from D2 (1.1-fold) to D5 (0.9-fold) and the NAC-treated group (Figure 3C and Supplementary Figure 1C) from D2 (1.2-fold) to D5 (1.2-fold) compared with the ISKN group from D2 (1.4-fold) to D5 (2.0); the results showed significant differences for both groups, up to 0.2-fold (D2) and 0.8-fold (D5), respectively, that were correlated with ROS generation, as shown in Figures 1, 2.

### Infectious spleen and kidney necrosis virus-induced reactive oxidative species stress signals can trigger cell death in fish cells

**FIGURE 4:**
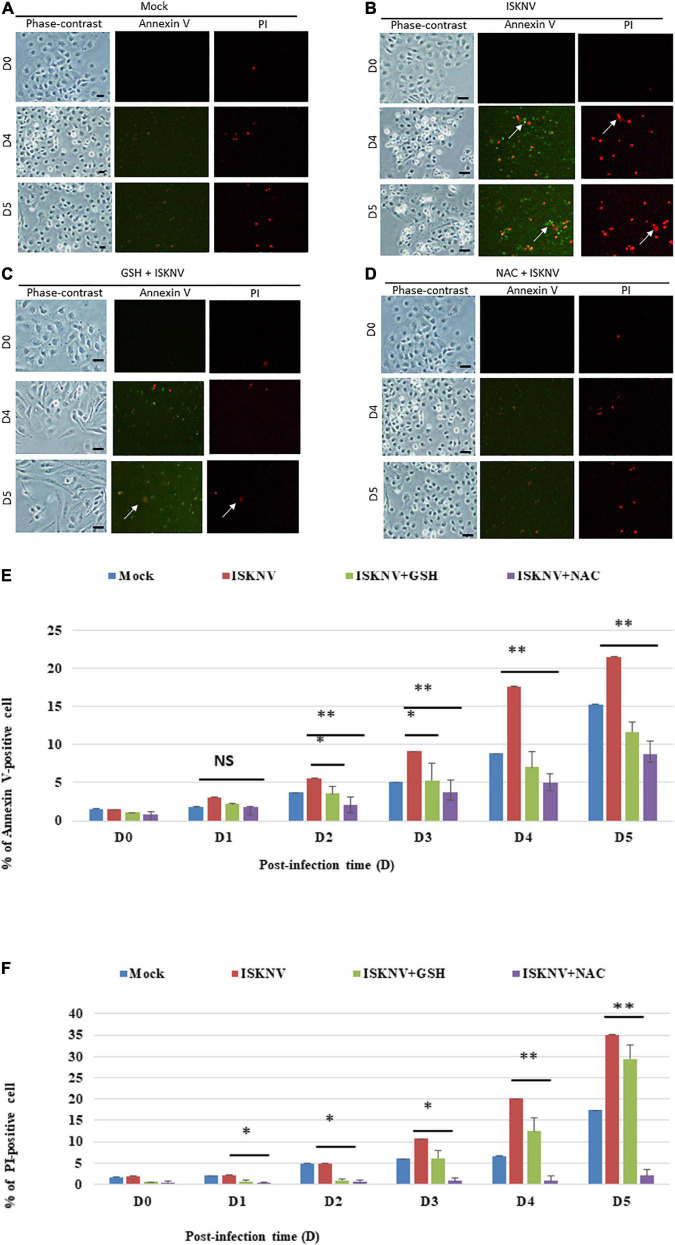
Glutathione (GSH)- and *N*-acetylcysteine (NAC)-blocked ISKNV induces apoptotic/necrosis cell death. Cells were labeled using annexin-V/PI staining [**(A–D)**: mock-control **(A)**, ISKNV-infected **(B)**, GSH-treated and virus-infected **(C)**, and NAC-treated and virus-infected **(D)**]. Phase contrast micrographs of ISKNV-induced apoptotic/necrotic fish cells at day 0, day 4, and day 5. Phase contrast images (left panels: day 0, day 4, and day 5), green fluorescence images for annexin-V-positive cells (middle panels: day 0, day 4, and day 5), and red fluorescence images for necrosis-positive cells (right panels: day 0, day 4, and day 5). Annexin-V-positive cells and necrosis-positive cells are indicated by arrows. **(E,F)** Percentage of apoptotic **(E)**/necrotic **(F)** ISKNV-infected cells at day 0, day 4, and day 5. The quantification of these results was performed for three individual experiments. All data were analyzed using either paired or unpaired Student’s *t*-tests, as appropriate. *P < 0.01 and **P < 0.05. ns, no significant different.

We further examined the inhibition of ISKNV-mediated stress signals by antioxidant treatment and cell death using an annexin-V/PI double-staining assay. A strong presence of apoptotic and necrotic cells was observed at D4 and D5 in the virus-infected cells when compared to the mock-control group, which was indicated either by green florescence or red florescence (Figure 4B), respectively (Figure 4A). As shown in Figures 4C,D for D4 and D5, respectively, the groups treated with GSH and NAC were found to suppress the apoptosis and necrosis rate more obviously than the ISKNV-infected group. We found the apoptosis and necrosis rate in the GSH-treated group was reduced by up to 7/7% and 12/12% at D4 and D5, respectively, and in the NAC-treated group was reduced by up to 12/16% and 20/19% at D4 and D5, respectively, when compared to the ISKNV-infected group at D4 (17.5/20%) and D5 (21.5/35%). The mock group as a normal control showed D4 values of 8.5/6.5% and D5 values of 15/18%.

### Blockage of Nrf2-mediated stress can suppress the mitochondrion-mediated Bax/Bak apoptotic cell death pathway in GF-1 cells

**FIGURE 5:**
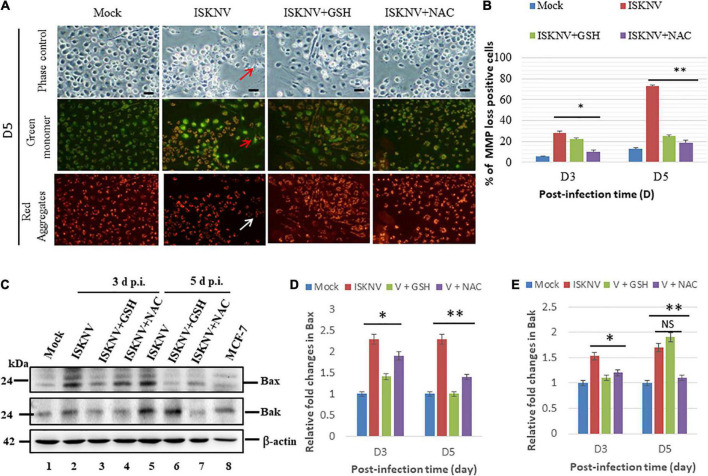
Glutathione (GSH) and *N*-acetylcysteine (NAC) can reduce infectious spleen and kidney necrosis virus (ISKNV)-induced mitochondrion-mediated Bax/Bak death signals in fish cells. **(A)** Identification of antioxidant-blocked ISKNV-induced MMP loss in fish cells at day 3 and day 5 post-infection. Phase contrast images for mock control group, ISKNV, GSH plus ISKNV, and NAC plus ISKNV (upper panels); green fluorescence images (middle panels) for MMP loss-positive cells; and red fluorescence images (lower panels) for MMP loss-positive cells, in which MMP loss-positive cells are indicated by arrows. Scale = 10 μm. **(B)** The ISKNV-induced losses of MMP in fish cells were counted at day 3 and day 5 post-infection. The quantification of these results was performed for three individual experiments. All data were analyzed using either paired or unpaired Student’s *t*-tests, as appropriate. *P < 0.01. **(C)** Identification of pro-apoptotic Bax and Bak proteins using western blot analysis. ISKNV-infected fish cells treated with GSH (2 mM) and NAC (2 mM) at day 0 (mock control), day 3, and day 5, where lanes 1–7 display ISKNV-infected fish cells and lane 8 shows the control MCF-7 cell lysate. Membranes were probed with anti-mouse Bax and Bak monoclonal antibodies (1:7,500) and an anti-mouse β-actin monoclonal antibody (1:12,500). **(D,E)** Quantification of Bax **(D)** and Bak **(E)** protein expression level (*N* = 3) using the ImageJ software, as shown in panel **(C)**. The data were analyzed using either paired or unpaired Student’s *t*-tests, as appropriate. Statistical significance was defined as *p-values < 0.05; ^**^p-values < 0.01 (Chen et al., 2022). ns, no significant different.

To determine whether ISKNV-mediated Nrf2 stress signals could trigger MMP loss, an evaluation was carried out using the MitoCapture reagent. In principle, JC1 dye (Chen et al., 2022) is trapped in mitochondria with normal ΔΨm in healthy cells and released from mitochondria with abnormal ΔΨm into the cytosol, leading to a loss of fluorescence intensity in apoptotic or necrotic cells. We found that Nrf2-mediated stress induced MMP loss (Figure 5A; indicated by arrows) and ISKNV-infected cells showed changes in the intensity of green and red fluorescence at D3 and D5, which showed losses between 30 and 71%, respectively, compared with the mock groups (Figure 5B). On the other hand, in the GSH-treated group, MMP loss was reduced by up to 7% (D3) and 45% (D5), and in the NAC-treated group, it was reduced by up to 13% (D3) and 52% (D5), showing the effective inhibition of MMP loss.

In addition, we further investigated the ISKNV-induced apoptotic cell death pathway in fish cells. We found that ISKNV infection induced the expression of the pro-apoptotic proteins Bax and Bak at D3 (Figure 5C, lane 2 and Supplementary Figure 2) and D5 (Figure 5C, lane 5 and Supplementary Figure 2). We also analyzed the effects of blocking Nrf2-mediated stress signals *via* GSH and NAC treatments on the pro-apoptotic genes Bax and Bak. According to the results, the upregulation of Bax and Bak was reduced in the GSH group at D3 (Figure 5C, lane 3 and Supplementary Figure 2) and in the NAC group at D3 and D5 (Figure 5C, lanes 4 and 7 and Supplementary Figure 2); however, an increase in Bak was not observed at D5 (Figure 5C, lane 6 and Supplementary Figure 2). Next, expression was quantified *via* densitometry using the relative fold changes shown in Figure 5D (Bax) and Figure 5E (Bak), which also showed up to a 0.5-fold change at D3 and D5 in both the GSH and NAC groups. Additionally, Bak suppression was shown to be very dynamic at D3, where its levels were reduced by up to 0.3-fold, while at D5, only the NAC group showed a 0.6-fold change, and Bak levels were not decreased in the GSH group.

### The antioxidant *N*-acetylcysteine inhibits caspase-9 and caspase-3 activation more effectively than glutathione

**FIGURE 6:**
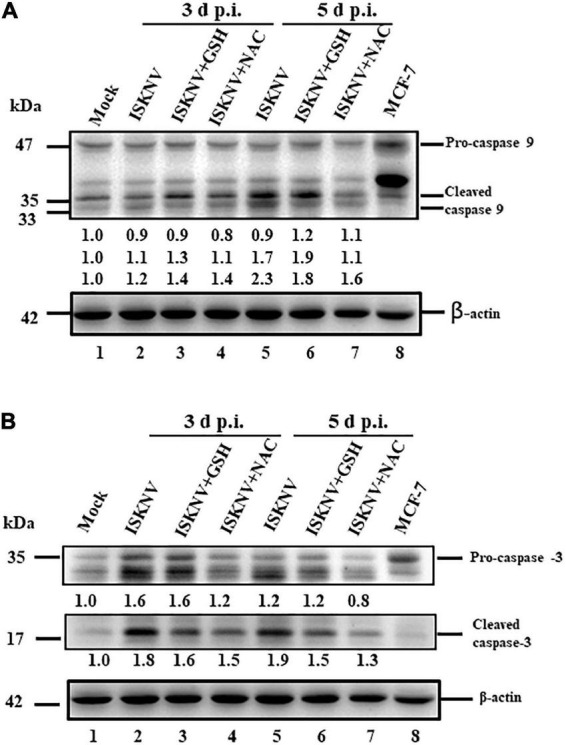
Glutathione (GSH) and *N*-acetylcysteine (NAC) can block infectious spleen and kidney necrosis virus (ISKNV)-activated caspases-9 and -3 in fish cells. **(A,B)** Infection of GF-1 cells with ISKNV and either GSH and NAC treatment at day 0 (mock control), day 3, and day 5, which blocked ISKNV-induced caspase-9 **(A)** and caspase-3 cleavage **(B)** from procaspase-9 and procaspase-3, as determined by western blot analyses. Lanes 1-6 correspond to ISKNV-infected fish cells at day 0, day 3, and day 5, while lane 8 shows the control MCF-7 cell lysate. Blots were probed with anti-mouse caspase-9 and caspase-3 monoclonal antibodies (1:7,500) and an anti-mouse β-actin monoclonal antibody (1:12,500). The quantification of protein expression levels was performed using the ImageJ software, as shown in panels **(A,B)**.

Next, we tried to answer the following question: can antioxidant drugs prevent cell death after the dual upregulation of positive death signals? The activation of caspase-9 and -3 has been implicated in the downstream death signaling of the mitochondrion-mediated pathway; here, we found that the activation of caspase-9 and -3 was blocked by cleaved products from pro-caspases that formed at D3 [Figures 6A,B, lanes 3 (GSH) and 4 (NAC) and Supplementary Figure 3] and D5 (Figure 6B, lanes 2). The changes from D1 (Figures 6A,B, lane 2 and Supplementary Figure 3) to D5 (Figures 6A,B, lanes 6 and 7 and Supplementary Figure 3) were analyzed using western blotting, and the results showed a correlation with the ROS/Nrf2-triggered mitochondrion-mediated cell death pathway, as shown in Figure 5.

## Discussion

Iridoviruses—in particular the ISKNV strain, which is found in a wide range of fish industries in the Asia Pacific region—have been scarcely examined in economically important fish, including marine fish and freshwater species (Williams et al., 2005; Kurita and Nakajima, 2012; Subramaniam et al., 2012). In this study, we examined ISKNV using a new approach and found that the virus triggers two ROS-mediated stress signals: signal one induces the pro-apoptotic Bax/Bak-mediated death pathway, which is correlated with the activation of the caspase-9/-3 pathway to trigger host cell death, while signal two induces the oxidative stress response *via* the expression of antioxidant enzymes, such as catalase, MnSOD, and Cu/ZnSOD, to reduce ROS generation during the viral replication cycle, which can suppress cell death and viral replication strength. Thus, we concluded that this novel examination of the ISKNV-induced pathogenesis will aid in its treatment and control.

### What kind of roles do reactive oxidative species-mediated stress signals play in host cells during viral infection?

Oxidative stress has been implicated in the pathogenesis of various neurodegenerative diseases, such as Alzheimer’s disease and Parkinson’s disease (Taylor et al., 2002; Jellinger, 2003). Oxidative stress occurs in cells when the production of reactive oxygen species (ROS) exceeds the cell’s endogenous antioxidant defenses (Krapfenbauer et al., 2003). In cells, the major defenses against ROS include superoxide dismutases (SODs) and catalase (Desagher et al., 1996; Dringen and Hampercht, 1997). SODs catalyze the dismutation of superoxide (O_2_^–^) to hydrogen peroxide (H_2_O_2_) and molecular oxygen (O_2_) and are located in the cytoplasm (Cu/ZnSOD) and mitochondria (MnSOD) (Schiavone and Hassan, 1988; Shull et al., 1991). Catalase is a tetrameric iron porphyrin protein found in the peroxisome that further scavenges H_2_O_2_ to produce H_2_O and O_2_ (McClung, 1997). The expression of catalase and Cu/ZnSOD is constitutive, whereas the expression of MnSOD within the mitochondria is induced by oxidative stress (Qi et al., 1997; Wang et al., 2003).

Many RNA viruses (Peterhans et al., 1987), DNA viruses (Vierucci et al., 1983), and retroviruses (Muller, 1992) can trigger oxidative stress and induce host cell death in infected cells. ISKNV-induced ROS production and its connection to pathogenesis have not been well-studied. Such studies may provide important insights into treatment.

In the present study, we found that ISKNV induced ROS production (Figure 1), causing an oxidative stress response that either triggered ROS/Nrf2-mediated signals (Figure 3) for antioxidant enzyme expression, helping the host to regulate metabolism when removing the ROS, or caused the upregulation of the expression of the pro-apoptotic genes *Bax and Bak* (Figures 5C–E), further damaging mitochondrial function by causing mitochondrial membrane potential (MMP; ΔΨ) loss (Figures 5A,B), which eventually culminates in host cell death.

### Is the reactive oxidative species/Nrf2 stress signal a common response during viral infection?

In recent years, it has been shown that ROS are important secondary messengers, and several sources of ROS, such as mitochondria, xanthine oxidase, NO synthase, and cytochrome P450 monooxygenases, have all been shown to be relevant in ROS production (Schroder, 2010). Complex I and complex III of the electron transport chain are the major sites of ROS production (Turrens and Boveris, 1980; Sugioka et al., 1988). The inhibition of complex I inhibition by rotenone has been shown to increase ROS generation in submitochondrial particles (Turens et al., 1985; Sugioka et al., 1988). In addition, the oxidation of either complex I or complex II substrates in the presence of complex III inhibition with antimycin A has been shown to increase the production of ROS (Turens et al., 1985; St-Pierre et al., 2002).

NADPH oxidases (Noxs) have recently received a great deal of attention. The family of NADPH oxidases consists of seven members, Nox1-Nox5, Doux1, and Doux2 (Sugioka et al., 1988), which all produce ROS. Different types of ROS are produced by the different NADPH oxidases. Nox4 predominantly generates hydrogen peroxide (H_2_O_2_), whereas superoxide anions (O_2_^–^) are produced by Nox1 and Nox2.

Recently, it has been shown that ROS can play a regulatory role in cellular metabolic processes *via* the activation of various enzymatic cascades, as well as transcriptional factors, that upregulate the expression of antioxidant enzymes, such as superoxide dismutase and glutathione peroxidase (Ott et al., 2007). In our system, ISKNV induced ROS production (Figure 1) at D1, and upregulated the transcription factor Nrf2 (Kaspar et al., 2009), catalase, Cu/ZnSOD, and MnSOD (Figure 3), which is a cellular sensor of chemical- and radiation-induced oxidative and electrophilic stress (Tilton et al., 2011; Lee, 2018) that controls the expression and coordinated induction of a battery of defensive genes encoding detoxifying enzymes and antioxidant proteins. Interestingly, in our system, ISKNV infection obviously upregulated Nrf2, Cu/ZnSOD, and catalase from D1 to D5 (Figure 3), which may have helped to restore ROS homeostasis. Furthermore, the antioxidants GSH and NAC (Figure 5) inhibited ISKNV-induced ROS production (Figure 2) and the induction of cell death (Figures 4, 5).

### Are reactive oxidative species/Nrf2 stress signals important in infectious spleen and kidney necrosis virus-induced Bax/Bak cell death?

A key step in the initiation of intrinsic apoptosis is mitochondrial outer membrane permeabilization (MOMP), which enables the release of pro-apoptotic factors, such as cytochrome *c*, from the mitochondrial intermembrane space (Tait and Green, 2010). The release of cytochrome *c* into the cytosol triggers the formation of the apoptosome, which induces the downstream activation of the initiator caspase-9 (Riedl and Salvesen, 2007; Tait and Green, 2010). Caspase-9 and caspase-8 can activate the downstream effector caspases, such as caspase-3 and -7. The BCL2 family proteins act as key regulators of intrinsic apoptosis by controlling mitochondrial outer membrane permeabilization (MOMP) (Youle and Strasser, 2008). The BCL2 family comprises pro- and anti-apoptotic members that share one or more BCL2-homology (BH) domains. Anti-apoptotic proteins (e.g., BCL2 and MCL1) contain four BH domains (BH1–4) and promote cell survival by antagonizing the activity of the pro-apoptotic BCL2 family members. The pro-apoptotic members can be divided into two subfamilies according to their BH domain composition: multi-domain pro-apoptotic proteins (e.g., BAK and BAX), which contain three BH domains, and “BH3-only” proteins (e.g., BAD, BID, BIK, and PUMA), which contain only the BH3 domain (Youle and Strasser, 2008; Shamas-Din et al., 2011). In our results, ISKNV infection was shown to upregulate the death factors Bax and Bak (Figure 5C and Supplementary Figure 2) at the middle–late replication stages. On the other hand, we found that caspase-9/caspase-3 were activated by precursors between D3 and D5 (Figures 6A,B and Supplementary Figure 3), while treatment with GSH and NAC blocked these signals effectively (Figures 4–6). Taken together, our results suggest that ROS/Nrf2-mediated signals are a key factor in controlling cell death related to infection with DNA or RNA viruses and may be good target molecules for therapy.

### Effects of stress signals on viral expression and replication

Recently, it was shown that ROS are important secondary messengers that can regulate viral replication. Few are currently known, especially in HBV (Daniela and Hildt, 2019), but this is a new era for the molecular pathogenesis of viral infections.

In our system, using a viral titer assay, we found that the ROS stress response in the late replication stage (at D5) could be reduced by approximately 1.2 log (GSH) and 2.0 log (NAC) after antioxidant treatment (Figure 6). Interestingly, at the late replication stage, the NAC-treated group was shown to have greater protection than the group treated with GSH; however, how viral replication may be regulated is still unclear and requires investigation.

**FIGURE 7:**
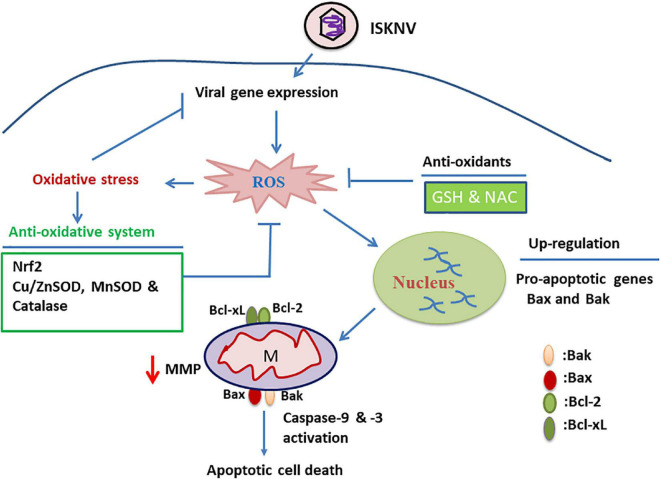
Hypothetical pathway where infectious spleen and kidney necrosis virus (ISKNV)-triggered reactive oxidative species (ROS)/Nrf2 stress signals either modulate the apoptotic Bax/Bak death pathway or reduce viral replication in fish cells. ISKNV shows a unique approach, using the ROS-mediated stress response that can either upregulate the death factor genes *Bax* and *Bak* or induce oxidative stress signals in the early viral replication stages in fish cells. In the ISKNV-induced Bax/Bak cell death pathway, these signals are also connected to changes in mitochondrial function, observed as MMP loss (ΔΨm) and caspase-9/-3 activation in fish cells. On the other hand, the oxidative stress response can also upregulate the stress transcriptional factor Nrf2 and antioxidant enzymes, such as catalase, Cu/MnSOD, and ZnSOD, which are correlated with the metabolism of ROS, such as superoxide and hydrogen peroxide, and thus the recovery of biological function in proteins or molecules. We found that the ISKNV-induced ROS/Nrf2-mediated Bax/Bak death pathway and viral replication can be inhibited by treatment with antioxidants such as GSH and NAC, providing a new strategy for viral control.

By comparing the results shown in Figures 1–4, 7, we found that ISKNV-induced ROS signals mildly modulated viral replication across all stages (D1–D5). During the replication stages, the ROS (hydrogen peroxide)-mediated response may play a major role in modulating the oxidative stress response to enhance viral replication for the upregulation of antioxidant enzymes, such as catalase or Cu/Zn SOD. However, it is still unclear how viral replication can be enhanced.

Summary (Figure 7 and Supplementary Figure 4): Taken together, our results suggest that novel ISKNV infection can trigger ROS/Nrf2-mediated stress signals that regulate apoptotic host cell death. The stress signals were correlated with upregulated Bax/Bak-mediated mitochondrial function loss, which triggered the activation of the death factors caspase-9 and -3. Finally, we found that ISKNV-triggered ROS/Nrf2 stress signals were blocked with GSH and NAC treatment, which either suppressed the Bax/Bak-mediated cell death pathway or modulated viral expression in the fish cells. These findings provide novel insights into large DNA viruses that use the host stress response to create a dynamic control strategy by which to regulate cell death and viral replication.

## Data availability statement

The raw data supporting the conclusions of this article will be made available by the authors, without undue reservation.

## Author contributions

J-RH: conceptualization, formal analysis, and writing—original draft preparation, review, and editing. P-HC and T-CH: methodology and investigation. All authors have read and agreed to the published version of the manuscript.
