# Supplementary figures and images for: Infectious spleen and kidney necrosis virus induces the reactive oxidative species/Nrf2-mediated oxidative stress response for the regulation of mitochondrion-mediated Bax/Bak cell death signals in GF-1 cells
Source: Front Microbiol. 2022 Oct 11;13:958476. doi: 10.3389/fmicb.2022.958476 (PMC9593061; doi:10.3389/fmicb.2022.958476)

Fig. 3A

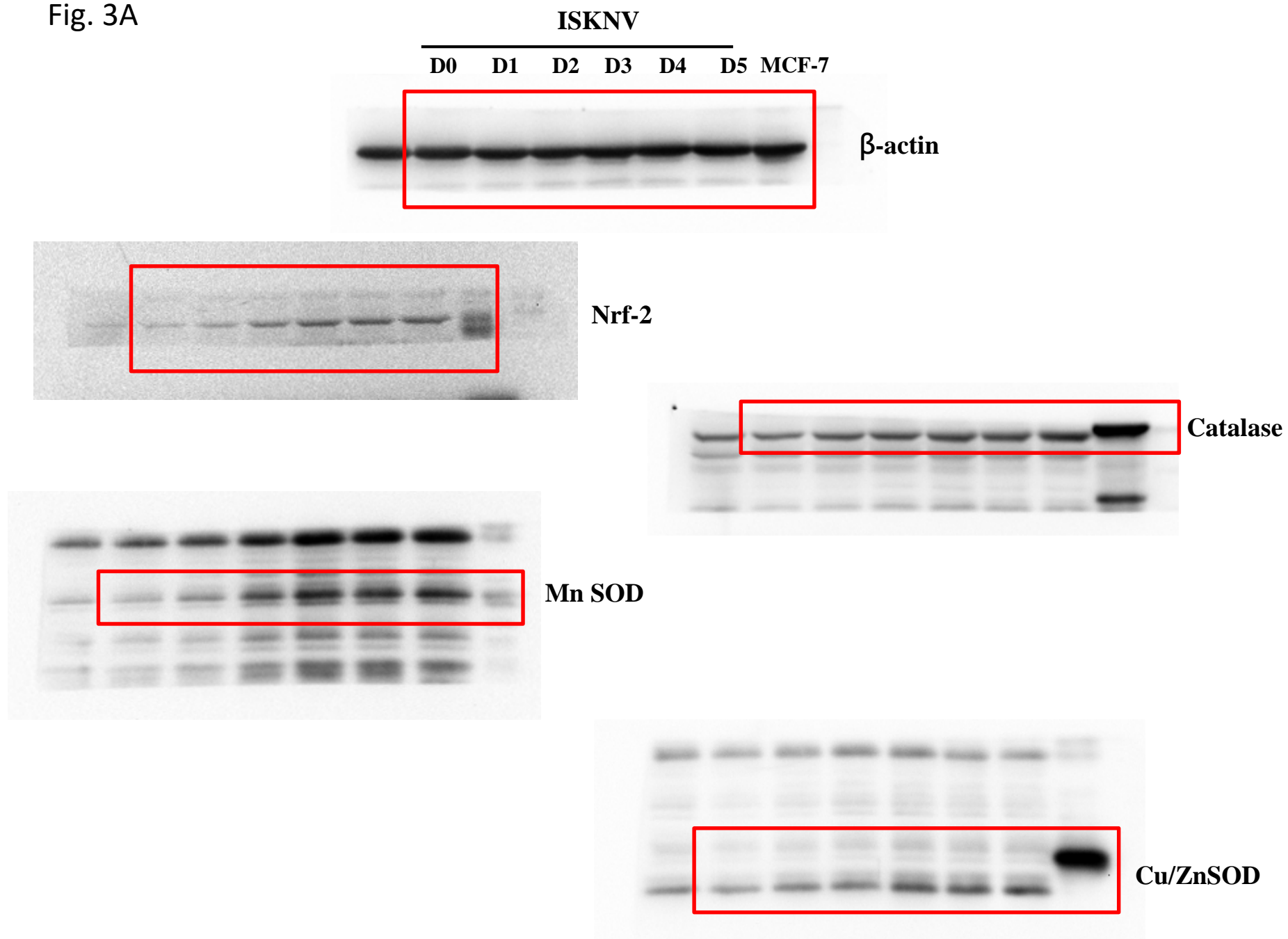

Fig. 3B

ISKNV + GSH

| D0 | D1 | D2 | D3 | D4 | D5 |
|----|----|----|----|----|----|
|----|----|----|----|----|----|

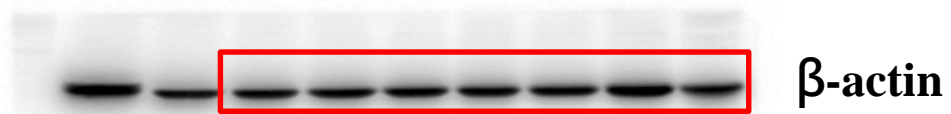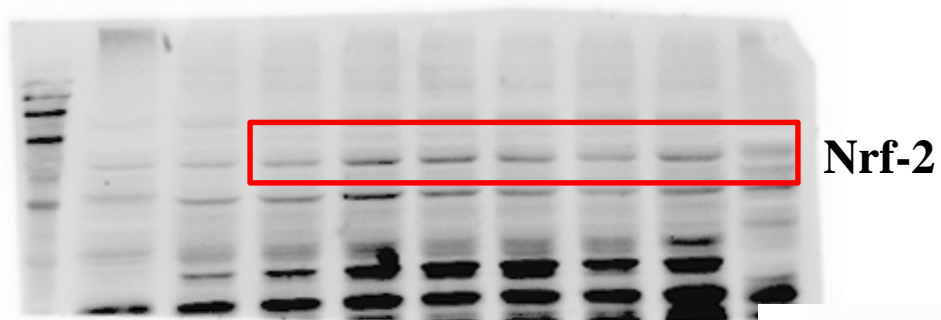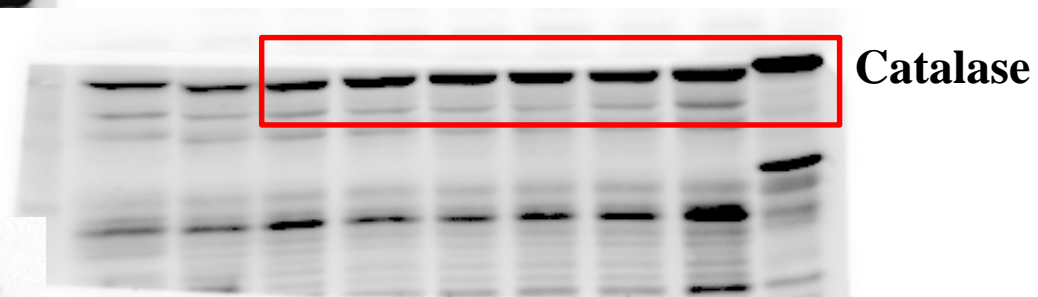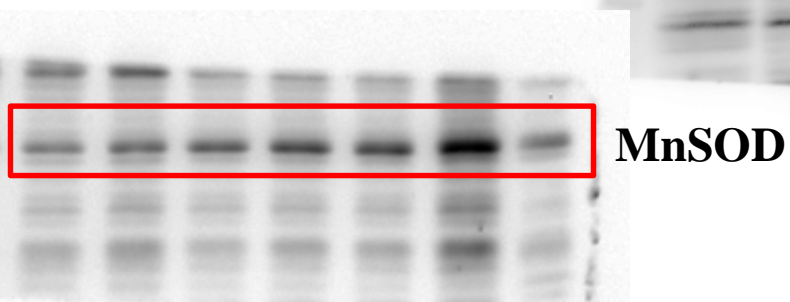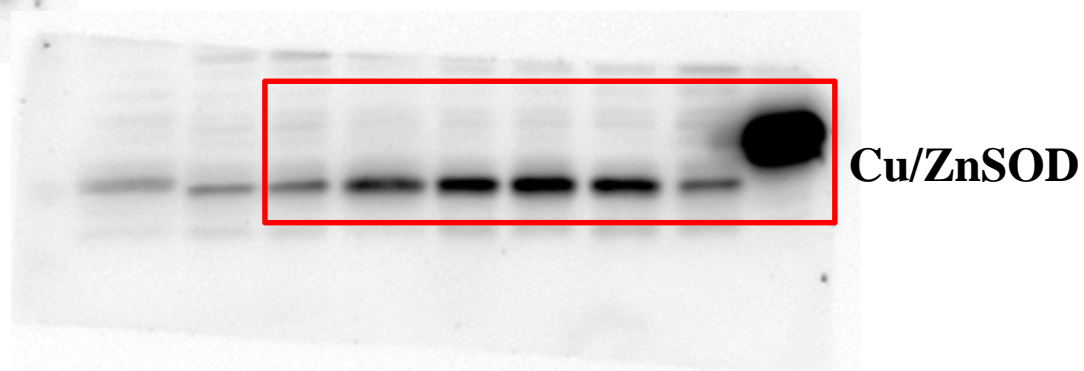

Fig. 3C

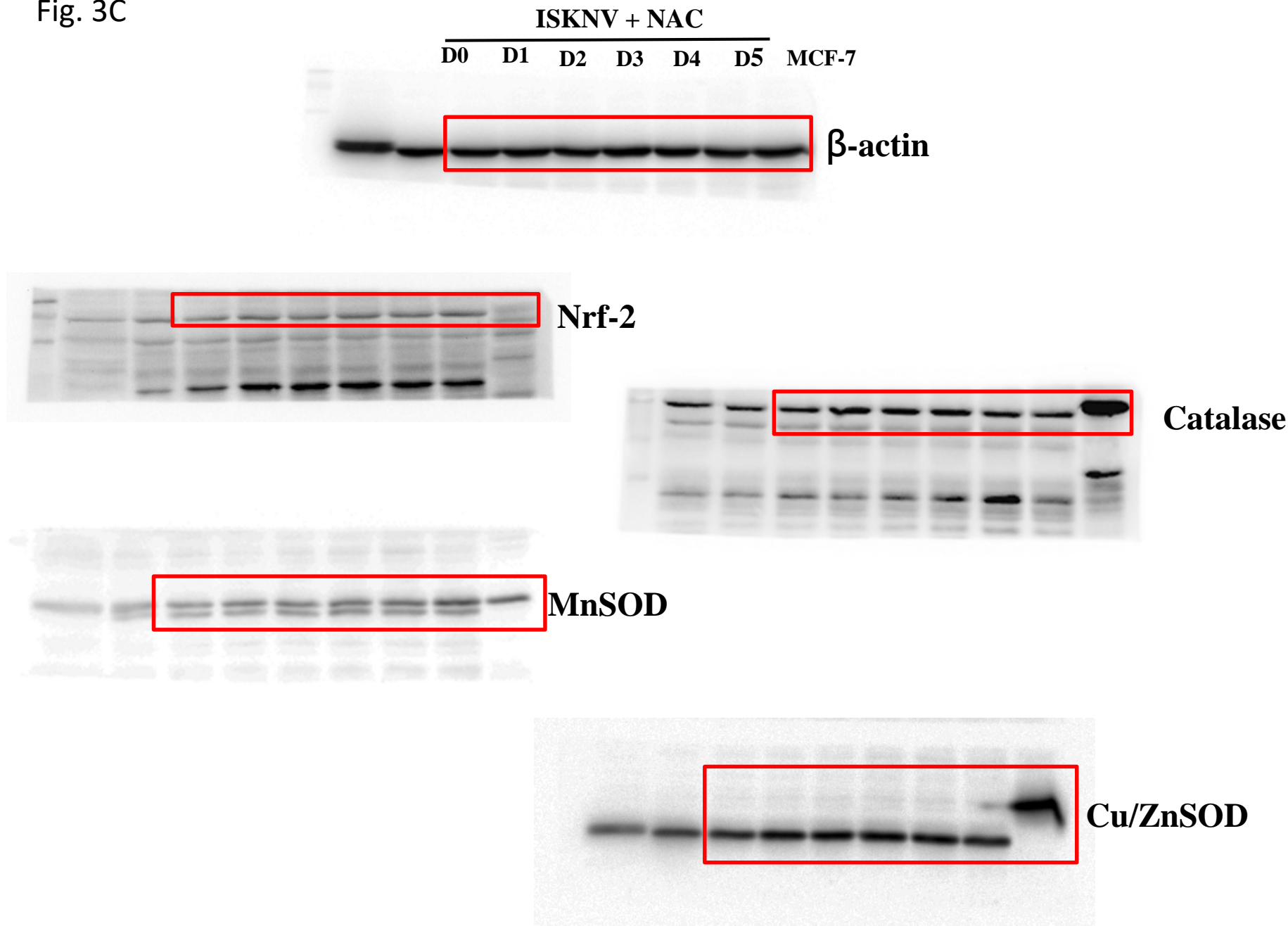

Fig. 5C

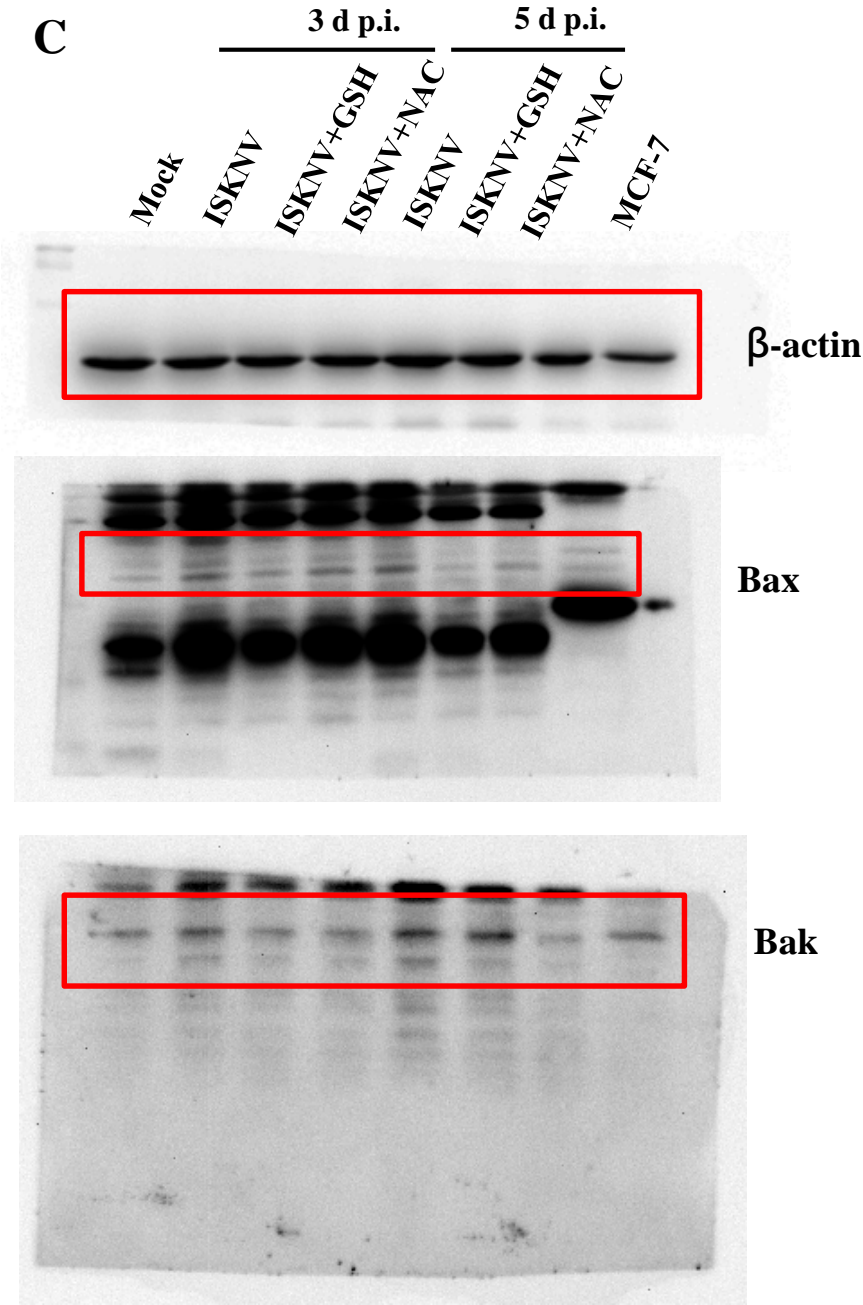

Fig. 6

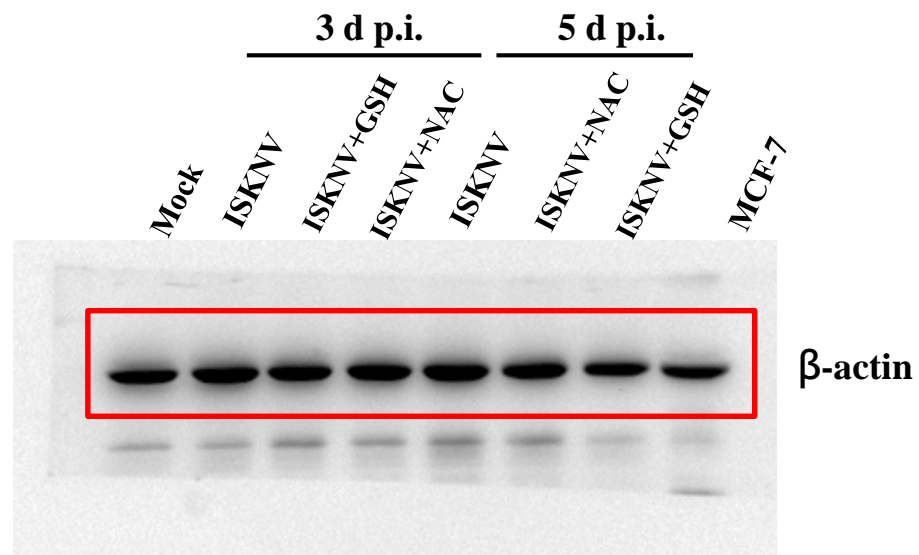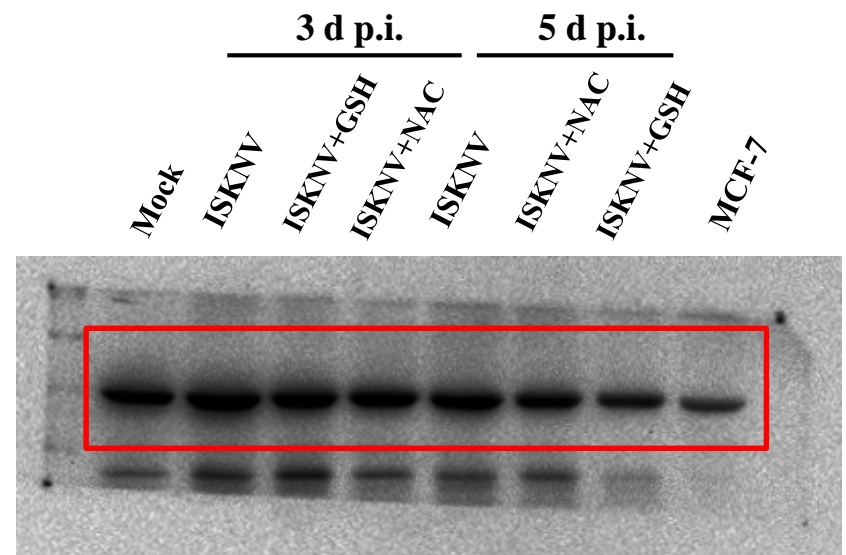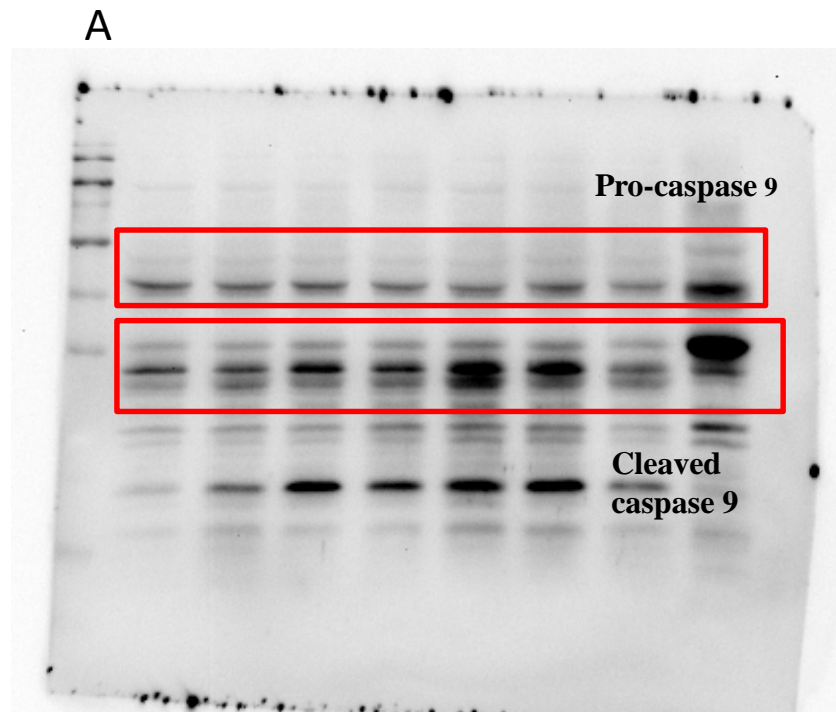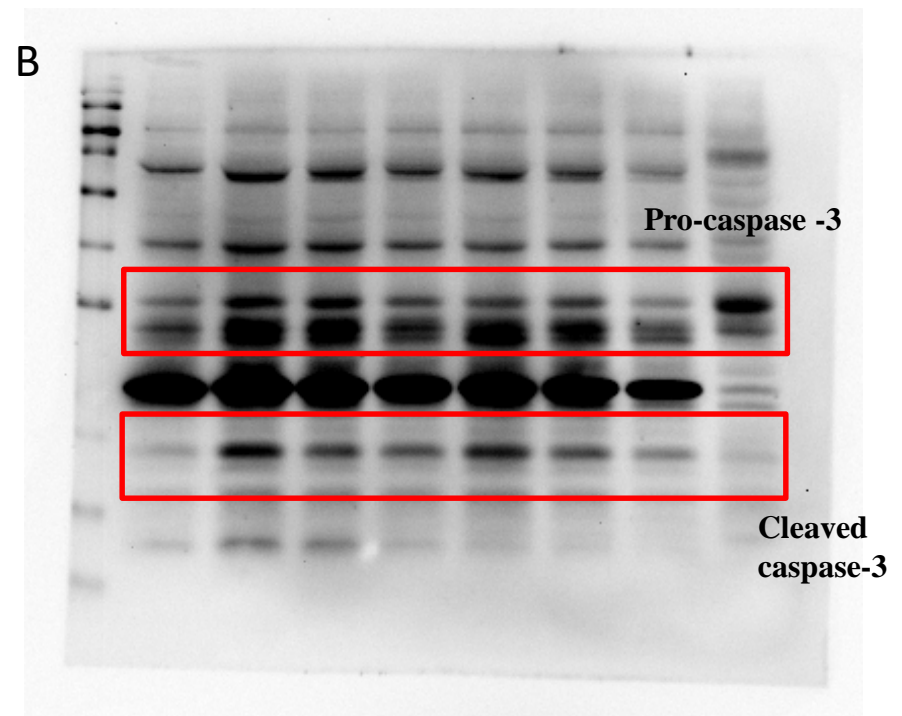

Fig. 7A

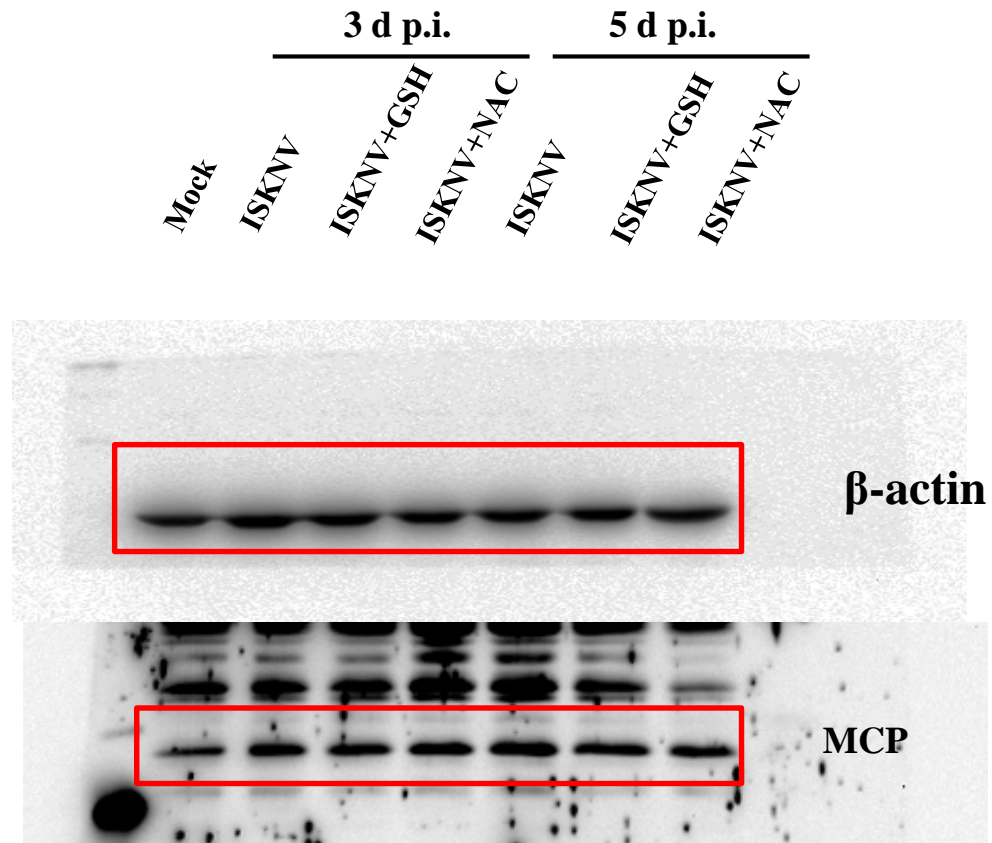

Supplement: Supplementary file 1 [file Data_Sheet_1.PDF]
